# Supplementary material for: Vulnerability Assessment of Pelagic Sharks in the Western North Pacific by Using an Integrated Ecological Risk Assessment
Source: Animals (Basel). 2021 Jul 21;11(8):2161. doi: 10.3390/ani11082161 (PMC8388413; doi:10.3390/ani11082161)
Supplement: Supplementary file 1 [file animals-11-02161-s001.zip › animals-1294458-supplementary.pdf]

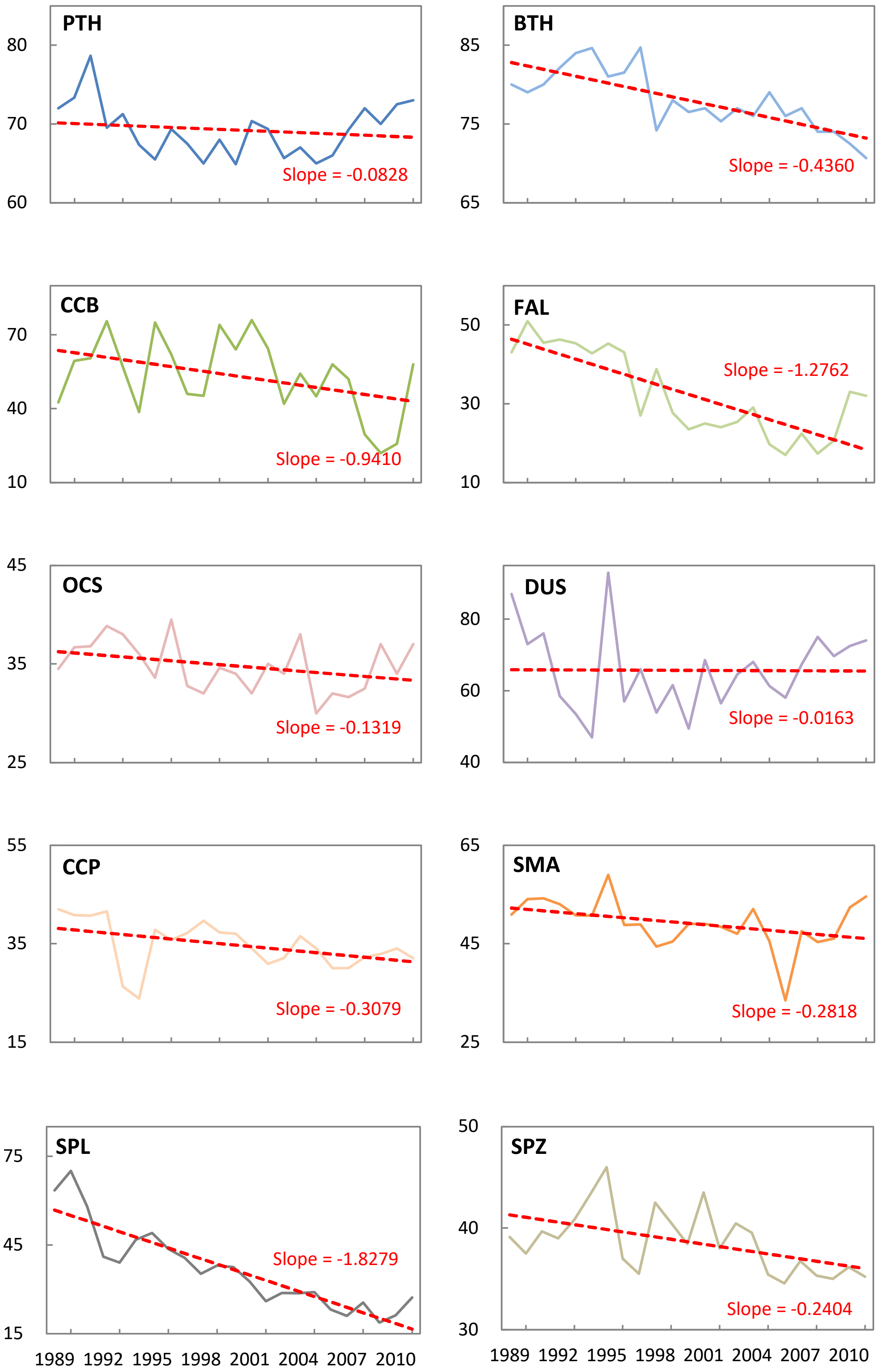

Supplement Figure S1. Annual median weight (kg) of 10 pelagic shark species landed at Nanfangao fish market from 1989 to 2011. PTH: pelagic thresher, *Alopias*

pelagicus; BTH: bigeye thresher, *A. superciliosus*; CCB: spinner, *Carcharhinus brevipinna*; FAL: silky, *C. falciformis*; OCS: oceanic whitetip, *C. longimanus*; DUS: dusky shark, *C. obscurus*; CCP: sandbar, *C. plumbeus*; SMA: shortfin mako, *Isurus oxyrinchus*; SPL: scalloped hammerhead, *Sphyrna lewini*; SPZ: smooth hammerhead shark, *S. zygaena*.
